# Determinants of stunting among children aged 6-59 months at Kindo Didaye woreda, Wolaita Zone, Southern Ethiopia: Unmatched case control study

**DOI:** 10.1371/journal.pone.0189106

**Published:** 2017-12-20

**Authors:** Bancha Batiro, Tsegaye Demissie, Yoseph Halala, Antehun Alemayehu Anjulo

**Affiliations:** 1 Kindo Didaye Woreda Health Office, Halale, Ethiopia; 2 School of Public Health, College of Health sciences and Medicine, Wolaita Sodo University, Wolaita Sodo, Ethiopia; 3 Department of Medical Laboratory, College of Health sciences and Medicine, Wolaita Sodo University, Wolaita Sodo, Ethiopia; University of Liverpool, UNITED KINGDOM

## Abstract

**Background:**

Stunting is a well-established risk marker of poor child development. Globally in 2017, 155 million children under 5 were estimated to be stunted. While different activities are being done to reduce the burden of stunted growth, the problem is overwhelming in Africa; it was increased by 24%. Therefore, identifying determinants of stunting among children aged 6–59 would help to set priorities for action and to the design of stunting reduction plan at a grassroots level.

**Methods:**

The unmatched case-control study was conducted in randomly selected 8 rural kebeles of Kindo Didaye woreda, Ethiopia from February to April, 2016 to identify the determinants of stunting among children aged 6–59 months. The sampling frame was identified by enumeration of 6–59 months of age children in the entire households of the study area. From which 155 as cases and 310 as controls were chosen using anthropometric measurement based on the median of WHO 2006 reference population. The anthropometric data were analyzed by WHO Anthro 2010 software to generate Z-score values. Odds Ratio along with 95% confidence interval was estimated to identify determinants of stunting using the multivariable logistic regression.

**Results:**

Drinking water from unsafe source (AOR = 7.06, 95% CI; 4.40–20.42),occasionally eating animal source food (AOR = 0.51, 95% CI; 0.02–0.68), ARI in the past two weeks (AOR = 3.04, (95% CI; 1.04–13.35), late initiation of breastfeeding after one hours after birth (AOR = 5.16, 95% CI; 2.24–15.90) and lack of vaccination (AOR = 6.38, 95% CI; 2.54–17.10)were significantly associated with stunting.

**Conclusions:**

Factors like exposure to diarrhea disease, exposure to acute respiratory infection, late initiation of breast milk after child breath, squeeze out of 1^st^ breast milk, lack of vaccination, animal source of food, and unsafe source of water for drinking could be used to set priorities for action and to the design of Kindo Didaye woreda plan for stunting reduction down to grassroots level. Therefore, zonal health department and Kindo Didaye woreda health office should promote the importance of colostrums feeding. Drinking water should be decontaminated. Expansion of vaccination program to enhance herd immunity at the community level is important.

## Introduction

Globally in 2017, 155 million children under 5 were estimated to be stunted. Childhood stunting is an outcome of maternal under nutrition and inadequate Infant and Young Child Feeding (IYCF), and would result in impaired neurocognitive development, and a risk factors for non-communicable diseases and reduced productivity in later life[1–2].

Its starts in the beginning of life and lasts throughout their life time [3]. An estimated 20% of stunting begins in the womb with a mother who herself is malnourished and is not getting enough of the nutrition she needs to support her baby’s growth and development during pregnancy. The estimated economic loss to Gross domestic Product (GDP) as result of stunting was 12%; and 75% of the world’s stunted children live in Sub Saharan Africa or Asia [4].

Stunting is measured by anthropometric indicator using height, sex and age in any stage of childhood of the child. Stunting is an indicator of linear growth retardation and cumulative growth deficits in children (Chronic malnutrition). Wasting measures body mass in relation to height and describes current nutritional status (acute malnutrition). Underweight takes into account both acute malnutrition (wasting) and chronic malnutrition (stunting), but it does not distinguish between the two [5–7].

According to 2015 Millennium development goals report, globally about 24.5% of children were stunted, 15% were underweight and 7.7% were wasted [8]. Stunting affecting more than161million children and one third of these were living in Africa [9]. A meta-analysis of demographic and health surveys (2006–2016) indicated that the prevalence of stunting varied in different part of Africa region and it’s high in East Africa region; Burundi (57.7%) and in Malawi (47.1%); in West Africa, Niger (43.9%), Mali (38.3%), Sierra Leone (37.9%) and Nigeria (36.8%); Democratic Republic of Congo (42.7%) and Chad (39.9%) in Central Africa[10]. A Tanzania study showed that the prevalence of stunting was 49.7% among under-five children [11]. The other cross-sectional household survey carried out to assess factors associated with stunting and severe stunting among under-fives in Tanzania showed that the prevalence of stunting and severe stunting were 35.5% and 14.4% for children aged 0–23 months and 41.6% and 16.1% for children aged 0–59 months, respectively[12].

In Ethiopia studies were conducted to assess the prevalence of stunting among under-five children. A community-based survey conducted in Shinille Woreda showed that the prevalence of stunting was 33.4% among children aged 6–59 months [13]. The other study carried out in East Belesa District, North West Ethiopia indicated that 57.7% of children were stunted [14]. The study conducted in Meskane Mareko District, Ethiopia showed that prevalence of stunting in the district was 43.7% among children aged 0–59 months [15]. Factors that contribute to stunted growth and development include: socio-economic and demographic factors like house-hold’s poverty, income, residence, occupation, education, maternal age, and family size [16].

While by 2025 World Health Assembly (WHA) target to achieve a 40% reduction in the number of under-five stunted children, the burden in Africa increased by 24% from 1990 to 2012. However, it was halved in Asia and Latin America/Caribbean [2, 17].

In June 2013, Ethiopia launched an ambitious and revised National Nutrition Plan (NNP) primarily focused on the first 1000 days; accelerated stunting reduction actions and multi-sectoral engagement in nutrition sensitive actions to transform the economic and development of millions of children and their mothers. The core performance indicators and targets to be achieved by 2015 were; reduce the prevalence of stunting from 44.4% to 30%, reduce the prevalence of wasting from 9.7% to 3% and reduce the prevalence of chronic under nutrition in women of reproductive age from 27% to 19% [18].

The importance of adequate nutrition is unquestionable but stunting extends beyond food availability and hunger, and has wide ranging consequences that prevent communities and nations from achieving their social and economic development aspirations. Thus, identifying determinants of stunting among children aged 6–59 months at Kindo Didaye woreda would help the health department to set priorities for action and to the design of stunting reduction plan at a grassroots level. Moreover, this study could add to the literature by providing additional information about determinants of stunting in the specific area.

## Methods

### Study area, design and period

A Community based unmatched case control study design was carried out from February to April, 2016 at Kindo Didaye woreda. Kindo Didaye woreda is one of the 12 woredas of Wolaita Zone in SNNPRS, Ethiopia; located at 86 kms far from the zonal capital Sodo and 475 Kms away from Addis Ababa. There are 22 kebeles in the woreda; of which 19 are rural administrative units and 3 are urban administrative unit with an estimated population of 120,446 as projected from 2007/2008 census. The numbers of children aged 6–59 months were 16,790 which account 13.94% of total population. The total area of the woreda is about 38045.7 hectares and sharing about 8.43% of the zonal area. There are three agro-ecological zones in the woreda in a range of 1200-to-2800 meter altitude above sea level [19].

The majority of Woreda (59%) classified as midland (‟woyndega”), 17.4% as high land and 23.6% as low land. The amount of average rainfall is about 1400mm-to- 2800mm per year. Economically the area is much dependant on agriculture and mostly kocho made from false banana (Enset ventricosum), kita (mainly maize) by cabbage and cassavas are common foods in the woreda. Cereals and roots are also common foods in the area. Crops like teff, wheat, barley and maize are common and sown often from May-July and harvested from September–November [19].

Regarding to a health facility, there were 25 health posts with two health extension workers at each health post, 04 health centers and 01 district hospital under government ownership providing health services for the community. Moreover, there were 04 medium clinics under private ownership [19].

### Source and study population

Source population were all children aged 6–59 months and their mothers /care giver`s resided in the woreda. Study population were all randomly selected children aged 6–59 months and their mothers/caregiver`s in selected kebele.

### Sample size calculation

The sample size was computed using STATCALC application of EPI- INFO version 7 software with the assumption: the percentage of children who were sick every month to be 14.6% for controls and of the cases 32.4%. 5% type I error, 80% power of the study, and case to control ratio of 1:2 to detect an odds ratio of 2.8[20–22], with 8% contingency for non-response rate and design effect of 2. The exposure variable was children who were sick every month [20]. Therefore, the required sample size was 465 (155 for cases and 310 for controls) (Table 1).

**Table 1:** Sample size determination by using factors associated with under-nutrition in Kindo Didaye woreda, Wolaita zone, southern Ethiopia, 2016.

Variables	Confidence level	Power	%of controls exposed	%of cases with exposure	Adjusted OR	Samplesize for bothgroups
Frequency of child’s illness(every month, once in two months, rarely>3months	95%	80%	14.6	32.4	2.8	216
Source of drinking water (unprotected, protected)	95%	80%	43.8	70.3	3.04	138
Children who had diarrhea before two weeks(Yes, No)	95%	80%	9.2	29.5	4.13	146

### Sampling procedures

A house to house census was made to enumerate 6–59 months of age children lived in 8 randomly selected kebele from the total 22 kebeles. All children aged 6–59 months and their mothers/care givers who lived for more than six months in the study area were enumerated. Anthropometric measurement of the children were taken to categorize the children as case or control based on the median of WHO 2006 reference population[23]. A child who had chronic malnutrition with z-scores ≤−2SD taken as a cases[10]and a child who had normal anthropometric reading with z-scores >-2SD to <+2SD (between -2SD and +2SD)and without bilateral pitting edema were taken as a control [21,24].

Totally 6,272 children aged 6–59 months were identified and registered sequentially and got identification number, 379 were enrolled as cases and 5893 as controls. One study subject was selected using lottery method if more than one eligible subject lived at a household level.

Study subjects were taken from randomly selected each kebele proportional to the number of sample size allocated for the study. A total of 465 study participants were selected. From this 155 were cases and 310 were controls. Finally, mother/care giver-child pairs from each selected kebele were enrolled using simple random sampling method and were followed. Moreover, kebeles were also selected using the following Formula.

ni = (n*Ni)/N where ni = sample size of each selected kebeles
n = total sample size
Ni = total number of children in each selected kebeles
N = total number of children in all selected kebeles

### Data collection procedures and instruments

Quantitative data were collected by qualified health care professional using pretested interviewer administered structured questionnaire (S1 File) from all eligible children mothers/care givers. A UNICEF recommended measuring instruments of wooden board inserted with a tape calibrated was used to collect the anthropometric data [25] from all children aged 6–59 months and their mothers/care givers who lived for more than six months in the study area, to identify retrospective morbidity of children, mothers were asked about any occurrence of illness during the past two weeks. Enumerators investigated to confirm nature and frequencies of illness based on operational case definition and also asked to identify occurrence of measles in the past one year. Vaccination status of children were checked by observing immunization card and if not available mothers /caregivers/ were asked to recall it. BCG vaccination was checked by observing scar on right (also left) arm.

#### Anthropometric measurement

Length/height was measured without shoes, socks, hair/head scurf, and ornaments and positioning the subject at the Frankfurt plane by using wooden board inserted with a tape calibrated to read the nearest 0.1cm. Length of the infants (6–23 months) was measured in a recumbent (lying) position using a horizontal wooden length board and movable headpiece. Height was measured in children older than two years of age in standing position into the nearest 0.1 cm using a vertical wooden height board by placing the child on the measuring board, and child standing upright in the middle of board and head held erect such that the external auditory meatus and the lower boarder of the eye were in one horizontal plane (Frankfurt plane). Anthropometric measurement was taken twice and a difference 0.1 cm in length was accepted as normal. However, repeated measurement was carried out upon significantly larger differences [24]. The child’s five contact points (head, shoulders, buttocks, knees and heels) were adjusted to touch the board [5].

### Data quality control

Training was given to the data collector and supervisors for two days prior to data collection in line with the objectives. Standardization test was made in 10 children before the actual survey and systematized based on the result to ensure the accuracy of anthropometric measurement. Two days before the actual study begins, the questionnaire was pre-tested and validated in 5% of mothers/care givers (not included in sample) selected from different kebele and some modifications were made based on response categories. Calibration of weight scale was also done before and after weighing every child by setting it to zero level with no object on it and placed in surface level before measurement of children.

### Data processing and analysis

Data were checked for completeness and entered into Epi Data Version 3.1 software. The entered data were then imported into SPSS version 20.0 for analysis. Hosmer–Lemeshow goodness-of-fit was used to test for the model fitness.

Anthropometric data were calculated by using WHO Anthro2010 software and height-for-age Z-scores were also been generated based on the median of WHO 2006 reference population (child growth standards)[26].Those children with Z-score value less than or equal to -2 standard deviations below median values of reference population were categorized as stunted.

In bivariate analysis, the variables that showed an association with the outcome variable with P- value <0.05 were entered into the multivariate model after checking multicollinearity to control for all possible potential confounders. According to Hosmer and Lemeshow’s recommendation on adequacy of sample size (155 stunted children) for logistic regression; the maximum numbers of variables (sixteen) were entered into the multivariable model [27].

Moreover, multicollinearity was checked using Variance Inflation Factor(VIF)and tolerance test. In this study, the maximum VIF became 1.86 while the tolerance value was less than 0.1, which proved the absence of multicolinearity between the independent variables. The p-value < 0.05 was considered to be significant and confidence interval for odds ratio was set at 95%.

### Ethical consideration

College of Health Sciences and Medicine Ethical committee at Wolaita Sodo University approved this study and provided ethical clearance letter. In addition to this, the university wrote letter of cooperation to Wolaita zone Health Department and Kindo Didaye woreda Health Office. Moreover, permission letter was obtained from kebele leaders in order to conduct the study in the local area. Written consent was obtained from each participant mothers/caregivers before starting the interview. Mothers/care givers who had children with Moderately Acute Malnutrition (MAM) and Severely Acute Malnutrition (SAM) lined with the nearest health facilities.

## Result

### Socio- demographic and economic characteristics of study participants

All study subjects were consented to participate and made the response rate 100%. The number of female were 65 (42%) and 150 (48.4%) in cases and controls groups, respectively. Majority of study subjects were Wolaita in both group; 154(99.4%) of cases and 305(98.4%) of controls. When we see religion; 86(55.5%) mothers of children in cases and 206(66.5%) in controls were protestant in religion. With regard to maternal education, 131(84.5%) of cases and 243(78.4%) of controls had illiterate mothers. Similarly, 136 (87.7%) of cases and 228(73.5%) of controls fathers were illiterate. Concerning mothers’ occupation, about 132 (85.2%) of the cases and 226 (72.6%) of the controls were housewife and similarly, fathers’ occupation 115 (74.2%) of cases and 146 (47.1%) of controls were farmers. In the family level fathers were responsible to use money in133 (85.8%) of cases house and 287 (92.6%) controls houses (Table 2).

**Table 2:** Socio-economic and demographic characteristics of households in Kindo Didaye woreda,Wolaita zone southern Ethiopia,2016.

Variables N = 465	Category	Cases No (%) N = 155	Controls No(%) N = 310
Religion	Protestant	86 (55.5%)	206(66.5%)
	Orthodox	69(44.5%)	101(32.6%)
	Others	0	3(0.9%)
Age of mothers	18–27	98(63.2%)	174(56.1%)
	28–37	42(27.1%)	128(41.3%)
	≥38	15(9.7%)	8(2.6%)
Marital status	Single	0	2(0.6%)
	Married	146(94.2%)	305(98.4%)
	Divorced	2(1.3%)	2(0.6%)
	Widowed	7(4.5%)	1(0.3%)
Educational status of mothers/care givers	No formal educt.	131(84.5%)	243(78.4%)
	Formal education	24(15.5%)	67(21.6%)
Occupation of mothers	House wife	132(85.2%)	226(72.9%)
	Employee	4(2.6%)	13(4.2%)
	Merchant	17(11%)	70(22.6%)
	Others	2(1.3%)	1(0.3%)
Number of family size	≤5	66(42.6%)	223(71.9%)
	>5	89(57.4%)	87(28.1%)
Number of under five children	≤2	87(56.1%)	287(92.6%)
	>2	68(43.9%)	23(7.4%)

### Maternal characteristics

The mean age ± standard deviation for the mothers of the cases was 30.25 (±4.97) years while it was 28.22 (±4.55) years for the mothers of the controls and which were between the ages of 28–37 years. Of the total 465 mothers/care takers interviewed during the study period, about 90(58.1%) and 217(70%) of mothers visited health facilities for antenatal care during their pregnancy period for both cases and controls respectively and from this 79 (51%) of cases and 159(51.3%) of controls had visit ≤3 times ANC follow up. On the other hand, 50(32.3%) and 191(61.6%) of mothers had taken extra food during their pregnancy period for both cases and controls respectively (Table 3).

**Table 3:** Obstetric factors children age 6–59 months in Kindo Didaye woreda,Wolaita zone southern Ethiopia, 2016.

Variable N = 465	Category	Cases No (%) N = 155	Controls No (%) N = 310
ANC follow up	No	65(41.9%)	93(30%)
	Yes	90(58.1%)	217(70%)
No of ANC follow up	≤3 Times	79(51%)	159(51.3%)
	4 Times	11(7.1%)	58(18.7%)
Counseling on breast complimentary feeding	No	3(1.9%)	4(1.3%)
	Yes	87(56.1%)	213(67.7%)
Counseling on feeding during pregnancy	No	10(6.5%)	5(1.6%)
	Yes	80(51.6%)	212(68.4%)
Extra food at pregnancy	No	40(25.8%)	26(8.4%)
	Yes	50(32.3%)	191(61.6%)
Extra food at lactation	No	27(17.4%)	12(3.9%)
	Yes	63(40.6%)	205(66.1%)
Frequency of breast feed within a day	<8 Times	90(58.1%)	43(13.9%)
	≥ 8Times	65(42%)	267(86.1%)

### Child characteristics

The mean age ± standard deviation for cases and controls were 29.4±15.5 and 30.3±15.2 months respectively, while their mean weight ± standard deviation for cases and controls were 10.43±2.48 and 12.88 ±2.45 kg respectively and also their mean height ± standard deviation for cases and controls were 84.38±11.76and 91.69±11.12cm respectively. Concerning under-nutrition in age category of total cases 19(12.3%), 44(28.4%), 36(23.2%), 36(23.2%) and 20(13%) belongs to age category 6–11, 12–23, 24–35, 36–47 and 48–59 months, respectively. Similarly, age category of total controls were 38(12.3%), 88(28.4%), 73(23.5%), 71(23%), 40(13%) belongs to age category 6–11, 12–23, 24–35, 36–47 and 48–59, respectively. Of the total 142(91.6%) mothers of cases and 266(85.8%) mothers of controls were delivered at home. 61(39.4%) of cases and 18(5.8%) of controls and 41(26.5%) of cases and 27(8.7%) of controls were the victim of diarrhea and acute respiratory infections in last two week respectively. However, 15(9.7%) of cases and 20(6.5%) of controls mothers helped their children to get treatment within 24 hours when their children manifested sign and symptoms of diseases (Table 4).

**Table 4:** Child characteristics among child aged 6–59 months in Kindo Didaye woreda,Wolaita zone southern Ethiopia, 2016.

Variables N = 465	Category	Cases No (%) N = 155	Controls No (%) N = 310
Sex of child	Male	80(51.6%)	160(51.6)
	Female	75(48.4%)	150(48.4)
Child age in months	6–11	19(12.3%)	38(12.3%)
	12–23	44(28.4%)	88(28.4%)
	24–35	36(23.2%)	73(23.5%)
	36–47	36(23.2%)	71(23%)
	48–59	20(13%)	40(13%)
Birth order of a child	1^st^	28(18.1%)	125(40.3%)
	2^nd^	71(45.8%)	152(49%)
	≥3^rd^	56(36.1%)	33(10.6%)
Birth interval	≤2	87(56.1%)	61(19.7%)
	>2	68(43.9%)	249(80.3%)
Diarrhea during last 2 weeks	No	94(60.6%)	292(94.2%)
	Yes	61(39.4%)	18(5.8%)
Time taken to health institutions for sick child	Within 24 hours	15(9.7%)	20(6.5%)
	>24hours	29(18.7%)	1(0.3%)
ARI	No	114(73.5%)	283(91.3%)
	Yes	41(26.5%)	27(8.7%)

### Child caring practices

From all mothers grouped under cases 66 (42.6%) and from all mothers grouped under controls 283(91.3%) started breast feeding within the 1^st^ one hour after birth. In addition to this, these mothers fed their child with colostrums immediately after birth. The exclusive breast-feeding rate for 6 months 32(20.6%) and 177(57.1%) for cases and controls respectively. Similarly, 123(79.4%) of cases and 133(42.9%) of controls were initiated complementary feeding before 6 months. Concerning immunization status 72(46.5%) of cases and 253(81.6%) of controls, 28(18.1%) of cases and 29(9.4%) of controls, 46(29.7%) of cases and 15(4.8%) of controls, 9(5.8%) of cases and 13(4.2%) of controls completed the vaccination according to WHO recommendation for his/her age, not completed, not at all vaccinated, and being vaccinating respectively. Seventy (45.2%) of cases and 57(18.4%) of controls were affected by worm infection, while from eligible children 63(40.6%) of cases and 166(53.5%) of controls received deworm per year (Table 5).

**Table 5:** Child caring practices of mothers/care givers of children aged 6–59 months in Kindo Didaye woreda,Wolaita zone southern Ethiopia, 2016.

Variables N = 465	Category	Cases No (%) N = 155	ControlsNo(%) N = 310
Breast milk initiated after delivery	Within the1^st^ one hour	66(42.6%)	283(91.3%)
	After 1 hour	89(57.4%)	27(8.7%)
Squeeze out of 1^st^ milk	No	66(42.6%)	282(91%)
	Yes	89(57.4%)	28(9%)
EBF duration in month	<6	123 (79.4%)	133(43%)
	At 6	32(20.6%)	177(57.1%)
Vaccination status	Completed recommended dose	72(46.5%)	253(81.6%)
	Not completed	28(18.1%)	29(9.4%)
	Not vaccinated	46(29.7%)	15(4.8%)
	Being vaccinating	9(5.8%)	13(4.2%)
Vitamin-A supplementation	No	35(22.6%)	29(9.4%)
	Yes	115(74.2%)	272(87.7%)
A child used fruits and vegetables occasionally	No	151(97.4%)	223(72%)
	Yes	4(2.6%)	87(28%)
A child used animal source of food occasionally	No	145(93.5%)	236(76.1%)
	Yes	10(6.5%)	74(23.9%)

### Environmental health condition of study participants

When we see latrine availability, 147(94.8%) households, from which cases were selected, had private latrine but only 76(49%) had fully functional hand washing materials at (near) toilet. Similarly, 303(97.7%) households, from which controls were selected, had private latrine but 88(28.4%) had fully functional hand washing materials at (near) toilet. Households used unsafe source of water for drinking accounts 82(52.9%) and 19 (6.1%) for both cases and controls respectively. Households with good hand washing practices was found to be 31(20%) in cases and 163(52.6%) in controls (Table 6).

**Table 6:** Environmental health condition of mothers/care givers of children aged 6–59 months in Kindo Didaye woreda,Wolaita zone southern Ethiopia, 2016.

Variables N = 465	Category	Cases No (%) N = 155	Control No(%) N = 310
Type of water source for drinking	Safe water	73(47.1%)	291(93.9%)
	Unsafe water	82(53%)	19(6.1%)
Latrine ownership(private)	No	8(5.1%)	7(2.3%)
	Yes	147(94.8%)	303(97.7%)
Functional hand washing facility near to the toilet	Full	76(49%)	88(28.4%)
	Partial	60(38.7%)	201(64.8%)
	Never at all	19(12.3%)	21(6.8%)
Hand washing practice	Mostly after Feeding	30(19.4%)	168(54.2%)
	Before food preparation	48(31%)	187(60.3%)
	After use of latrine	151(97.4%)	308(99.4%)
	After cleaning child	43(27.7%)	201(64.8%)
	As mentioned all	31(20%)	163(52.6%)

### Determinants of stunting among children aged 6–59 months

Most variables that were associated with stunting during bivariate analysis were lost their significance in the multivariable model and only the following seven variables(unexposed to diarrhea disease, acute respiratory infection, initiation of breast milk after one hours after child breath, squeeze out of 1^st^ breast milk, lack of vaccination, animal source of food, and unsafe source of drinking water) retained their significance in the multivariable model with P-value < 0.05 and considered as significant and independent predictors of stunting.

According to this study, study subjects unexposed to diarrhea disease in the past two weeks prior to data collection were 80% (AOR = 0.20, 95% CI; 0.05–0.90) less likely to be stunted when we compared with children exposed to diarrheal diseases. Children with ARI in the past two weeks prior to the data collection were 3 times (AOR = 3.04, (95% CI; 1.04–13.35) more likely to be stunted as compared to children who had no history of ARI in the past two weeks prior to the data collection.

This study also showed that initiation of breastfeeding after one hour after birth had statistically significant association with stunting. Initiation of breastfeeding after one hour after child birth were about 5 times (AOR = 5.16, 95% CI; 2.24–15.90) more likely to be stunted as compared to initiation of breastfeeding within one hour after child birth. Besides this, squeezed out of first breast milk was showed statistically significant association with outcome variable. Those children whose mothers squeezed out of first breast milk following delivery were 5.7 times (AOR = 5.72, 95%CI; 2.58–16.14) more likely to be stunted as compared to those children’s whose mothers give first breast milk just following delivery.

This study demonstrated that, children vaccination status was showed statistically significant association with stunting. Children who were not vaccinated his/her recommended vaccine dose for his/her age were 6.38 times (AOR = 6.38, 95% CI; 2.54–17.10) more likely to be stunted as compared with children who received appropriate dose of vaccine.

Animal source of food had statistically significant association with stunting. Study subjects who ate animal source food were 49% (AOR = 0.51; 95% CI: 0.02–0.68) less likely to be stunted than who did not eat. Moreover, Source of drinking water also had statistically significant association with stunting. Individuals whose families use drinking water from unsafe source were about 7 times (AOR = 7.06, (95% CI; 4.40–20.42) more likely to be stunted as compared to those who used safe source (Table 7).

**Table 7:** Determinants of stunted among children aged 6–59 months in Kindo Didaye woreda, 2016.

Variable N = 465		Nutritional status		COR(95% CI)	AOR(95% CI)
		Cases N = 155	Control N = 310		
**Educational status of father**					
Formal education		136(87.7%)	228(73.5%)	1.00	1.00
No formal education		19(12.3%)	82(26.5%)	2.57(1.32–9.71)	1.70(0.64–4.47)
**Family size:**	≤ 5	66(42.6%)	223(72%)	1.00	1.00
	>5	89(57.4%)	87(28.1%)	0.29(0.15–0.95)	0.75(0.28–1.99)
**Under-5 child size:**	≤ 2	87(56.1%)	287(92.6%)	1.00	1.00
	>2	68(43.9%)	23(7.4%)	0.10(0.08–0.92)	0.56 (0.15–2.03)
**Priority to food distribution in HH during feeding:**					
Child		50(32.3%)	193(62.3%)	1.00	1.00
Father		38(24.5%)	35(11.3%)	4.19(2.40–7.29)	0.80(0.18–3.45)
Mother		0	3(1%)		
Both jointly		67(43.2%)	79(25.5%)	3.27(2.08–5.13)	0.77(0.17–3.36)
**ANC follow up**	No	65(42%)	93	1.68(1.39–5.83)	7.82(0.25–9.35)
	Yes	90(58.1%)	217	1.00	1.00
**Extra food at pregnancy**	No	40(25.8%)	26	5.87(3.27–10.53)	0.90(0.19–4.19)
	Yes	50(32.3%)	191	1.00	1.00
**Extra food at lactation**	No	27(17.4%)	12	7.32(3.50–15.28)	4.86(0.85–27.61)
	Yes	63(40.6%)	205	1.00	1.00
**Birth interval**	≤2	87(56.1%)	61	5.22(3.42–7.97)	3.27(0.98–10.86)
	>2	68(43.9%)	249	1.00	1.00
**Diarrhea during last 2 weeks**	No	94	292	1.00	1.00
	Yes	61	18	0.09(0.05–0.17)	0.20 (0.05–0.90)*
**ARI during last 2 weeks:**	No	114	283	1.00	1.00
	Yes	41	27	0.26(0.15–0.45)	3.04 (1.04–12.35)*
**Time at which breast milk initiated:**					
Within the1^st^ 1 hour		66	283	1.00	1.00
After 1 hour		89	27	0.07(0.04–0.11)	5.16(2.24–15.90)**
**Squeeze out of 1^st^ milk**	No	60	280	1.00	1.00
	Yes	95	30	0.07(0.04–0.12)	5.72(2.58–16.14)***
**Vaccination status:**					
Completed recommended dose		72	253	1.00	1.00
Not completed		28	29	3.39(1.90–6.07)	0.70(0.19–2.53)
Not at all vaccinated		46	15	10.77(5.68–20.41)	6.38(2.54–17.10)**
Being vaccinating		9	13	2.43(1.02–5.92)	0.55(0.02–13.50)
**A child used animal source of food occasionally:**					
No		145	236	1.00	1.00
Yes		10	74	4.5(2.27–9.08)	0.51(0.02–0.68)*
**Type of water source for drinking:**					
Safe water		73	291	1.00	1.00
Unsafe water		82	19	0.06(0.03–0.10)	7.06(4.40–20.42)***

**N.B**

*(P<0.05).

** (P<0.01).

*** (P<0.001).

1.00 = reference category.

## Discussion

This study pointed out that determinants of stunting were exposure to diarrheal disease, exposure to acute respiratory infection, late initiation of breast milk after child breath, squeeze out of 1^st^ breast milk, lack of vaccination, animal source of food, and unsafe source of drinking water.

Exposure to diarrheal diseases had significant association with stunting. The finding of this study was similar with the other studies conducted in different parts of the world like Ethiopia, Nepal, Vietnam and Afghanistan [20–22, 28–31]. All the referenced studies confirmed that exposure to diarrhea had association with under-nutrition. This might be because of loss of fluids and electrolytes, loss of food appetite and absorption in the intestine.

Exposure to Acute Respiratory Infections (ARI) in the past two weeks prior to the data collection was found to be the risk factors for stunting. Study subjects with ARI were 3 times more likely to be undernourished as compared to those without the complaint of ARI. This was similar with the other studies conducted in Ethiopia and India [29, 32–34]. The reason for this could be both respiratory system and gastro intestinal system among children covered by human healthy microbiota like Lactobacilli and Bifidobacteria usually obtained from breast milk. Children acquire these microorganisms via breast milk and these agents help them as an immune barrier and to facilitate re absorption in gastro intestinal tract among children [35]. Frequent infection alters the normal flora like lactobacilli and then children become susceptible to infectious agents and loss re absorption in the intestine.

According to this study late initiation of breastfeeding usually after one hour after birth had statistically significant association with under- nutrition. Individuals started breastfeeding after one hour after births were 5 times more likely to be stunted as compared to children started breastfeeding within one hour after birth. This result was in agreement with the other study conducted in Tigray region of Ethiopia [36]. This could be due to early initiation resulted in increased production of milk but late initiation of breastfeeding was associated with decreased newborn-mother bonding and then inadequate maternal breast milk secretion.

In this study, squeeze out of first milk was shown to be predictors of stunting. Those children whose mothers Squeezed out of first breast milk following delivery were 5.7 times more likely to be undernourished as compared to those children’s whose mothers give first breast milk just following delivery. This finding was similar with the other studies conducted in different part of Ethiopia, West Gojjam and Machakel Woreda of Ethiopia. Both studies concluded that squeezed out of colostrums was more likely to be undernourished than those who fed colostrums [20,37]. This might be because of majority of mothers involved in this study were illiterate and they had poor awareness about the importance of providing colostrums to their children. Mothers living in the study area had the habit of squeeze out of first milk. Because, it is thought that feeding of colostrums affect infant health status and interferes with breastfeeding in the community. However, colostrums’ is three times richer in vitamin A and ten times richer than in beta-carotene than mature milk. Due to their high levels of vitamin A, antibodies, and other protective factors, colostrums is often considered as the baby’s first immunization [38]. But, mothers who have the habit of squeeze out of first milk denying colostrums and their baby becomes at risk for stunting.

In terms of vaccination, children who did not receive any vaccine were 6.38 times more likely to be under-nourished as compared to those who received with appropriate dose of vaccine.This finding was consistent with the study conducted in Gimbi district and Machakel Woreda of Ethiopia [20, 39]. This probably due to unvaccinated children might face to some of the infectious diseases like pneumonia, diarrhea and measles. So, their children might have been exposed to under-nutrition.

According to this study individuals who ate occasionally from animal source of food were protected from being stunted than who did not eat. This result was in line with the other similar studies conducted in Wondo Genet of Ethiopia and India [29, 34]. The reason for this might be animal source of food have various health benefits and good source of many nutrients with readily absorbable and convenient form; which helps for production of immunity which facilitates health status of children.

Other important predictor that was associated with nutritional status of the children was source of drinking water. Those children whose families use drinking water from unsafe source were 7 times more likely to be undernourished as compared to those who used safe source. This finding agreed with studies conducted in Ethiopia [32, 36], Nigeria [40], and Guatemala [20, 41]. All the referenced studies concluded that access to unsafe water supply has been found as a risk factor for chronic under-nutrition. This might be because of unsafe water aggravates the spread of water-borne diseases (like repeated diarrheal disease and intestinal parasites) that can affect the health and nutritional status of children. This study could be subjected to recall bias; Mothers/caregivers were interviewed in retrospect. The other limitation could be measurement bias that happened during Anthropometric measurement of the children.

## Conclusions

Factors like exposure to diarrhea disease, exposure to acute respiratory infection, late initiation of breast milk after child breath, squeeze out of 1^st^ breast milk, lack of vaccination, animal source of food, and unsafe source of water for drinking could be used to set priorities for action and to the design of Kindo Didaye woreda plan for stunting reduction down to grassroots level.

Therefore, Zonal health department and health office should work aggressively to prevent and reduce stunting at a grassroots level.

## Supporting information

S1 File English version survey questionnaire. (DOCX)

## References

[1] WHO. Infant and young child feeding. July 2017 /www.who.int/mediacentre/factsheets/fs342/en/.accessed date September 2, 2017

[2] WHO, Childhood Stunting: Challenges and opportunities Report of a Promoting Healthy Growth and Preventing Childhood Stunting colloquium. Geneva: World Health Organization; 2014

[3] Getabil, F. Prevalence of and Factors Associated With Stunting among Under-Five Children in Ethiopia;2012.

[4] /https://thousanddays.org/the-issue/stunting/ accessed date 10 January 2016.

[5] Tamiru Mekides Wondemeneh, Belachew Etana Tolessa, and Abera S.F. Under Nutrition and Associated Factors Among Under-Five Age Children of Kunama Ethnic Groups in Tahtay Adiyabo Woreda, Tigray regional State, Ethiopia: Community based study. International Journal of Nutrition and Food Sciences, 2015 4 (3): p. 278–289.

[6] Agency, E.C.S. and O. Macro. Ethiopia Demographic and Health Survey. Addis Ababa, Ethiopia and Calverton. Maryland: Central Statistical Agency and ORC Macro 2006.

[7] De Onis M. WHO child growth standards: length/height-for-age, weight-for-age, weight-for-length, weight-for-height and body mass index-for-age WHO; 2006

[8] United Nations. The Millennium Development Goals Report. 2015.

[9] UNICEF, WHO, and T.W. Bank. Joint Child Malnutrition Estimates,Levels & Trends in Child Malnutrition. 2014.

[10] Akombi BJ, Agho KE, Merom D, Renzaho AM, Hall JJ.Child malnutrition in sub Saharan Africa: Ameta-analysis of demographic and healthsurveys(2006–2016). PLoSONE 2017;12(5):e0177338. 10.1371/journal.pone.0177338 PMC5426674 28494007

[11] Semali et al Prevalence and determinants of stunting in under-five children in central Tanzania:remaining threats to achieving Millennium Development Goal 4. BMC Public Health 2015; 15:1153 doi: 10.1186/s12889-015-2507-6 26590803 10.1186/s12889-015-2507-6 PMC4654796

[12] Chirande et al Determinants of stunting and severe stunting among under-fives in Tanzania: evidence from the 2010 cross-sectional household survey. BMC Pediatrics 2015; 15:165 doi: 10.1186/s12887-015-0482-9 26489405 10.1186/s12887-015-0482-9 PMC4618754

[13] Maalin et al Magnitude and factors associated with malnutrition in children 6–59 months of age in Shinille Woreda, Ethiopian Somali regional state: a cross-sectional study. BMC Nutrition 2016; 2:44

[14] Fentahun et al Under nutrition and associated factors among children aged 6–59 months in East Belesa District, northwest Ethiopia: a community based cross-sectional study. BMC Public Health 2016;16:506 doi: 10.1186/s12889-016-3180-0 27297078 10.1186/s12889-016-3180-0 PMC4906918

[15] Hagos S, Hailemariam D, WoldeHanna T, Lindtjørn B. Spatial heterogeneity and risk factors for stunting among children under age five in Ethiopia: A Bayesian geo-statistical model. PLoS ONE 2017; 12(2): e0170785 doi: 10.1371/journal.pone.0170785 28170407 10.1371/journal.pone.0170785 PMC5295674

[16] Egata Gudina, Berhane Yemane, and Worku A. Predictors of acute undernutrition among children aged 6 to 36 months in east rural Ethiopia: a community based nested case—control study. BioMed Central Ltd., 2014: p. 14:91. 10.1186/1471-2431-14-91 PMC3994202 24708711

[17] UNICEF-WHO-World Bank Joint child malnutrition estimates. New York: UNICEF;Geneva:WorldHealthOrganization;WashingtonDC:TheWorldBank;2012,updated2013. (http://apps.who.int/gho/data/node.main.ngest?lang=en accessed date September 10 2016)

[18] (17/UNICEF Ethiopia—Nutrition—Nutrition.htm)

[19] Office, K.D.W.A.R.D., Annual report. 2014.

[20] Bantamen G., Belaynew W., and Dube J. Assessment of factors associated with malnutrition among under five years age children at Machakel Woreda, Northwest Ethiopia: a case control study. Journal of Nutrition & Food Sciences, 2014 4(1): p. 1.

[21] Wong H.J., Moy F.M., and Nair S. Risk factors of malnutrition among preschool children in Terengganu, Malaysia: a case control study. BMC public health, 2014 14(1): p. 785. 25086853 10.1186/1471-2458-14-785 PMC4133606

[22] Dereje N. Determinants of Severe Acute Malnutrition among Under Five Children in Shashogo Woreda, Southern Ethiopia: A Community Based Matched Case Control Study. Nutrition and Food Sciences, 2014 4(5).

[23] Onis D. and Mercede. WHO child growth standards: length/height-for-age, weight-for-age, weight-for-length, weight-for-height and body mass index-for-age. 2006: WHO.

[24] Egata Gudina, Berhane Yemane, and Worku A. Predictors of acute undernutrition among children aged 6 to 36 months in east rural Ethiopia: a community based nested case—control study. BioMed Central Ltd., 2014: p. 14:91. 10.1186/1471-2431-14-91 PMC3994202 24708711

[25] Mengistu Kebede, Alemu K., and Destaw B. Prevalence of malnutrition and associated factors among children aged 6–59 months at Hidabu Abote District, North Shewa, Oromia Regional State. J Nutr Disorders Ther, 2013 1: p. 1–15.

[26] WHO, Multicentre Growth Reference Study Group: WHO Child Growth Standards based on length/height, weight and age. Acta Paediatr 2006,450:76–85.]. 10.1111/j.1651-2227.2006.tb02378.x 16817681

[27] John Willey& Sons. Applied Logistic Regression. Thrid Edition. 2013:, Inc.

[28] Solomon Amsalu and Z. Tigabu. Risk factors for severe acute malnutrition in children under the age of five: A case-control study. Ethiopian Journal of Health Development, 2008 22(1): p. 21–25.

[29] Ayana A.B., Hailemariam T.W., and Melke A.S. Determinants of acute malnutrition among children aged 6–59 months in Public Hospitals, Oromia region, West Ethiopia: a case–control study. BMC Nutrition, 2015 1(1): p. 1.

[30] Yisak Hiwot, Gobena Tesfaye, and F. Mesfin. Prevalence and risk factors for under nutrition among children under five at Haramaya district, Eastern Ethiopia. BMC Pediatrics 2015: p. 15:212 doi: 10.1186/s12887-015-0535-0 26675579 10.1186/s12887-015-0535-0 PMC4682239

[31] Frozanfar M.K., et al Acute malnutrition among under-five children in Faryab, Afghanistan: prevalence and causes. Nagoya J. Med. Sci., 2016 78: p. 41 ~ 53. 27019527 PMC4767513

[32] Demissie Solomon and Worku A. Magnitude and Factors Associated with Malnutrition in Children 6–59 Months of Age in Pastoral Community of Dollo Ado District, Somali Region, Ethiopia. Science Journal of Public Health, 2013 1(4): p. 175–183.

[33] Basit A., et al Risk factors for under-nutrition among children aged one to five years in Udupi taluk of Karnataka, India: A case control study. Australasian Medical Journal 2012 5, 3: p. 163–167. doi: 10.4066/AMJ.20121022 22952561 10.4066/AMJ.20121022 PMC3433731

[34] Woldie Yadessa Tegene, et al Prevalence of Stunting and Associated Factors among Under Five Children In Wondo Genet Woreda, Sidama Zone, Southern Ethiopia. International Journal of Medical and Health Sciences Research 2015 2(2): p. 36–49.

[35] Soto A. et al Lactobacilli and Bifidobacteria in Human Breast Milk: Influence of Antibiotherapy and Other Host and and Clinical Factors. J Pediatr Gastroentrol Nutr. 2014;59(1):78–88. 10.1097/MPG.0000000000000347 PMC4086764 24590211

[36] Alemayehu Mussie, et al Nutritional status and associated factors among under-five children, Tigray, Northern Ethiopia. International Journal of Nutrition and Food Sciences 2014 3(2327–2694): p. 3(6): 579–586

[37] Beka T, et al Magnitude and determinants of stunting in children under five years of age in food surplus region of Ethiopia: The case of West Gojam Zone. Ethiopian Journal of Health Development, 2009; p.23: 2.

[38] http://www.lalecheleague.org/faq/colostrum.html. accessed date September 2016,

[39] E, K. Prevalence and determinants of child malnutrition in Gimbi district, Oromia region, Ethiopia faculty of medicine, community health department Addis Ababa. 2007.

[40] OB R. et al Prevalence and Determinants of Malnutrition among Under-five Children of Farming Households in Kwara State, Nigeria: Canadian Center of Science and Education. Journal of Agricultural Science 2011: p. 3(3).

[41] Macro and ORC. Central Statistical Agency Addis Ababa, Ethiopia. 2011.

