# Supplementary material for: Determinants of stunting among children aged 6-59 months at Kindo Didaye woreda, Wolaita Zone, Southern Ethiopia: Unmatched case control study
Source: PLoS One. 2017 Dec 20;12(12):e0189106. doi: 10.1371/journal.pone.0189106 (PMC5737969; doi:10.1371/journal.pone.0189106)
Supplement: S1 File — (DOCX) [file pone.0189106.s001.docx]

## English version survey Questionnaire

English version Survey questionnaire to identify predictors to under-nutrition among 6-59 months of children in rural community of Kindo Didaye woreda, Wolaita zone, southern Ethiopia

**Study Identification**

| 001. | Name of woreda  ________________ | 007 | Time of interview  Started_____________  Completed___________ |
| --- | --- | --- | --- |
| 002. | Name of kebele  ________________ | 008 | Number of visits to HHs by data collectors Sign |
| 003 | Kebele Id No |  |  |
| 004 | Name of sub kebeles (Got) | 009 | Name of interviewer’s Name of interviewer  Sign |
| 005 | Got(village) Id No | 010 | Id No of interviewer’s |
| 006 | participant’s Id__________________ | 011 | Name of supervisor  Sign |
|  |  | 012 | Id No of supervisor |

**Visiting Table**

|  | Visit 1 | Visit 2 | Visit 3 |
| --- | --- | --- | --- |
| Date |  |  |  |
| Result* |  |  |  |

*Result code 1- Complete

2- Incomplete/partially completed

3- Respondent not available

4- Other, specify_______ _______

**Part I**: Socio- demographic and economic characteristics of households

| S.No | Question | | | | Response | | | | Code | | remark | | | |
| --- | --- | --- | --- | --- | --- | --- | --- | --- | --- | --- | --- | --- | --- | --- |
| 101 | What is your ethnicity? | | | | 1.Wolaita  2.Gamo  3.Other(specify)_________ | | | |  | |  | | | |
| 102 | What is mother’s/care giver`s age?  (in years) | | | |  | | | |  | |  | | | |
| 103 | What is your religion? | | | | 1. Protestant  2. Orthodox  3. Catholic  4.Other(specify)_________ | | | |  | |  | | | |
| 104 | What is your current marital status? | | | | 1. Single  2. Married  3. Divorced  4. Widowed | | | |  | |  | | | |
| 105 | | Did you ever attend formal education | | | 1. Yes 2. No | | | |  | |  | | | |
| 106 | | Your husband did attend formal education? | | | 1. Yes 2. No | | | |  | |  | | | |
| 107 | | What is your occupation? | | | 1. House wife  2. Employee  3. Merchant  4. Other(specify)______ | | | |  | |  | | | |
| 108 | | What is the occupation of your husband? | | | 1. Farmer  2. Employee  3. Merchant  4.Other(specify)______ | | | |  | |  | | | |
| 109 | | What is the total number of your family size that present currently? | | |  | | | |  | |  | | | |
| 110 | | What is the total number of under- five children in your home? | | |  | | | |  | |  | | | |
| 111 | | Who is your household head? | | | 1.Your husband  2. Yourself  3. Other(specify)________ | | | |  | |  | | | |
| 112 | | Does this household own livestock? | | | 1.Yes  2.No | | | |  | |  | | | |
| 113 | | How much is average monthly income of your household in ETB? | | |  | | | |  | |  | | | |
| 114 | | Does this household have own agricultural land? | | | 1.Yes  2.No | | | |  | |  | | | |
| 115 | | If yes, how many oxen of agricultural land ?(Ask them how many oxen)  (4oxens = 1 hectare= 1000^m2^) | | |  | | | |  | |  | | | |
| 116 | | Who have taking priority to food distribution in household during feeding? | | | 1. Father  2. Mother  3. Child  4. Both jointly | | | |  | |  | | | |
|  | | | | |  | | | |  |  |  |  |  |  |
|  | | | | |  |  |  |  |  |  |  |  |  |  |
|  | | | | | | | | | | | | | | |
| **Part II: Maternal characteristic** | | | | | | | | | | | | | | |
| 201 | Did you visit health facility for ANC during your Pregnancy for this child? | | | | | 1. Yes 2. No |  | | | If No 208 | | |  |  |
|  |  |  |  |  |  |  |  | | |  |  |  |  |  |
| 202 | If yes how many times did you visit during time of your pregnancy of this child?( No of follow up) | | | | |  |  | | |  | | |  |  |
| 203 | Did you get counseling on child feeding (breast and Complimentary feeding) during ANC visit of this child? | | | | | 1.Yes  2. No |  | | |  | | |  |  |
| 204 | Did you get counseling on how you must feed yourself during the ANC visit? | | | | | 1.Yes  2. No |  | | | If No 208 | | |  |  |
| 205 | If yes, did you receive extra food during pregnancy? | | | | | 1. Yes 2. No |  | | |  | | |  |  |
| 206 | If yes for Q204, did you receive extra food during lactation | | | | | 1.Yes  2. No |  | | |  | | |  |  |
| 207 | How many times did you feed breast within a day (24hrs)? | | | | |  |  | | |  | | |  |  |
| **Part III: Child characteristics** | | | | | | | | | | | | |  |  |
| 301 | | | Child sex | 1. Male 2. Female | | | |  | | | |  | |  |
| 302 | | | Child age in months  (verify child`s date of birth by seeing the child’s health card or use local calendar) | Months | | | |  | | | |  | |  |
| 303 | | | Date (day, month and year) of child birth? (ask and write) |  | | | |  | | | |  | |  |
| 304 | | | Where did you deliver this child? |  | | | |  | | | |  | |  |
| 305 | | | Gestational age at birth?  (ask and write) |  | | | |  | | | |  | |  |
| 306 | | | Types of birth (ask and write) |  | | | |  | | | |  | |  |
| 307 | | | What was the size of this child according to your observation? |  | | | |  | | | |  | |  |
| 308 | | | Birth order of this child(ask and write) |  | | | |  | | | |  | |  |
| 309 | | | What is birth interval b/n the youngest and his/her immediate elder in years? |  | | | |  | | | |  | |  |
| 310 | | | Did the child have diarrhea during last 2 weeks? | 1. Yes 2. No | | | |  | | | |  | |  |
| 311 | | | Did the child have ARI during last 2 weeks? | 1. Yes 2. No | | | |  | | | |  | |  |
| 312 | | | Did the child have measles in the past one year? | 1. Yes 2. No | | | |  | | | |  | |  |
| 313 | | | When the child sick at what time ever taken to health institutions? |  | | | |  | | | |  | |  |
| 314 | | | Height of a child | Cm | | | |  | | | |  | |  |
| **Part IV-Child caring practices** | | | | | | | | | | | | | |  |
| \| 401 \| Time at which breast milk initiated after delivery \| 1. Within the1^st^ hour 2. After 1 hour 3. If other(Specify) \|  \|  \|  \| \| --- \| --- \| --- \| --- \| --- \| --- \| \| 402 \| Squeeze out of 1^st^ milk \| 1. Yes 2. No \|  \|  \| \| 403 \| If yes, why? \| 1.Not important  2. Lack of awareness \|  \|  \| \| 404 \| How long did you exclusively breast feed this child? \| Months \|  \|  \| \| 405 \| Child age at which you initiated complementary feeding for this child \| Months \|  \|  \| \| 406 \| Did a child have worm infection? \| 1. Yes 2. No \|  \|  \| \| 407 \| Did a child receive de-worming? \| 1.Yes  2. No \|  \|  \| \| 408 \| If yes, how many times a child received de-worming? \|  \|  \|  \| \| 409 \| Did a child receive Vitamin A supplementation? \| 1. Yes  2. No \|  \|  \| \| 410 \| Vaccination status of children (Check immunization card, scar) \| 1. Completed recommended for his age 2. Not Completed 3. Not at all vaccinated 4. Being vaccinating \|  \|  \| \| 411 \| Your child feed on fruits and/or Vegetables occasionally? \| 1.Yes  2. No \|  \|  \| \| 412 \| Your child feed on animal source of food occasionally?  (Especially milk, poultry and meat their derivatives) \| 1. Yes 2. No \|  \|  \|   **Part V: Environmental health condition**   \| 501 \| What type of water source are you used for drinking? \| 1. Protected spring water  2. Unprotected spring water  3. Public stand pipe(tap)  4. Other(specify) \|  \|  \|  \| \| --- \| --- \| --- \| --- \| --- \| --- \| \| 502 \| Latrine ownership(private) \| 1. Yes  2. No \|  \|  \| \| 503 \| Is there functional hand washing facility at (near) toilet? (Check it) \| 1. Full 2. Partial 3. Never at all \|  \|  \| \| 504 \| What was your hand washing practice? \| 1. After cleaning child  2. Before food preparation  3. Mostly before feeding  4. After use of latrine  5. As mentioned all \|  \|  \| | | | | | | | | | | | | | |  |
